# Mitochondrial Respiration in Intact Peripheral Blood Mononuclear Cells and Sirtuin 3 Activity in Patients with Movement Disorders

**DOI:** 10.1155/2017/9703574

**Published:** 2017-09-10

**Authors:** Slawomir Michalak, Jolanta Florczak-Wyspiańska, Joanna Rybacka-Mossakowska, Wojciech Ambrosius, Krystyna Osztynowicz, Aleksandra Baszczuk, Wojciech Kozubski, Ewa Wysocka

**Affiliations:** ^1^Department of Neurochemistry and Neuropathology, Poznan University of Medical Sciences, Przybyszewskiego str. 49, 60-355 Poznan, Poland; ^2^Department of Neurology, Poznan University of Medical Sciences, Przybyszewskiego str. 49, 60-355 Poznan, Poland; ^3^Chair and Department of Laboratory Diagnostics, Poznan University of Medical Sciences, Szamarzewskiego str. 82/84, 60-569 Poznan, Poland

## Abstract

**Objective:**

Mitochondrial dysfunction is considered a unifying pathophysiological explanation for movement disorders. Sirtuin 3 (SIRT3) exhibits deacetylase activity and antioxidant properties. The aim of the study was to analyze the mitochondrial respiration in peripheral blood mononuclear cells (PBMCs) and the SIRT3 activity in patients with movement disorders.

**Methods:**

Mitochondrial respiration was analyzed in intact PBMCs using the ROUTINE, LEAK, electron transfer system (ETS), and residual oxygen consumption (ROX) protocol by means of high-resolution respirometry. The SIRT3 expression and PBMC activity were measured using fluorometry. Ultrasound measurements of the echogenicity of the substantia nigra and the diameter of the 3rd ventricle were also performed.

**Results:**

Patients with movement disorders exhibited a lower ROUTINE respiration than controls (*P* = 0.0237). Reduced oxygen fluxes in the LEAK (*P* = 0.033) and ROX (*P* = 0.0486) states were observed in patients with movement disorders compared with controls. Decreased ROUTINE respiration (*P* = 0.007) and oxygen flux in the LEAK state (*P* = 0.0203) were observed in patients with PD with substantia nigra hyperechogenicity compared with controls. Decreased SIRT 3 deacetylase activity was found in patients with movement disorders.

**Conclusion:**

Impaired mitochondrial respiration in intact PBMCs was associated with inhibited SIRT3 activity and neurodegeneration measures evaluated using ultrasound in patients with PD.

## 1. Introduction

Movement disorders constitute a group of degenerative conditions in the central nervous system, including primarily Parkinson's disease (PD), atypical parkinsonian syndromes, dystonia, essential tremor, and restless legs syndrome. The idiopathic form of PD affects 1% of the population over 65 years of age with the incidence of 8–18 cases per 100,000 persons per year. With a prevalence of approximately 3% in persons over 75 years of age, neurodegenerative diseases are confirmed to develop predominantly in the elderly and are a major cause of disability in this population [1].

In contrast to idiopathic PD, other movement disorders like progressive supranuclear palsy (PSP, Steele-Richardson-Olszewski's disease), corticobasal degeneration (CBD), multisystem atrophy (MSA), and diffuse Lewy body disease (DLBD) were considered atypical parkinsonism (AP).

Mitochondrial dysfunction was suggested as a unifying pathophysiological explanation for PD and other neurodegenerative disorders. Mitochondria are the most efficient producers of adenosine triphosphate (ATP) and play an important role in calcium homeostasis and in the initiation of apoptosis. In the case of severe oxidative phosphorylation defects, the ability of mitochondria to handle calcium is impaired [2]. Moreover, mitochondria are a major source of reactive oxygen species (ROS), including manganese superoxide dismutase (Mn-SOD, SOD2), and promote defense mechanisms against ROS-mediated damage. Hence, mitochondrial and oxidative stress are implicated in a number of neurodegeneration and aging-related disorders. Genes associated with autosomal recessive PD are reported to be involved in the control of the mitochondrial function. Phosphatase/tensin homolog deleted on chromosome 10- (PTEN-) induced putative kinase 1 (*PINK1*) and *Parkin* genes controls the elimination of impaired mitochondria via autophagy (mitophagy) [3].

Phosphorous magnetic resonance spectroscopy revealed impairments in the mitochondrial function in patients with PSP. Moreover, oxidative metabolism in the brain and skeletal muscles of patients with PSP was also reduced [4].

Movement disorders associated with alpha-synuclein (*α*-syn) pathology (PD, MSA, DLBD) are classified as synucleinopathies, while others associated with tau pathology like PSP and CBD are classified as tauopathies.

The role of *α*-syn in the pathophysiology of PD seems to be dichotomous and awaits further elucidation. This small 14-kDa (140 amino acids) presynaptic protein, with a propensity to aggregate into oligomers in a prion-like manner [5] and to form intraneuronal protein inclusions called Lewy bodies, can participate in defense mechanisms preserving the neural mitochondrial homeostasis against oxidative stress, without protecting against stressors directly affecting mitochondrial function [6]. Mutations in the *SNCA1* gene, which encodes *α*-syn, cause familial PD and are risk factors for sporadic PD [7]. Accumulation of *α*-syn aggregates in neurons is a key process in the PD pathogenesis. These aggregates are found not only in the substantia nigra pars compacta in PD but also in other neurons in the central and peripheral nervous system [8, 9]. There are conflicting reports on the role of *α*-syn in the production of proinflammatory cytokines in microglia or monocytes [10–13]. The expression of *α*-syn in peripheral blood mononuclear cells (PBMCs) was found to be upregulated in idiopathic PD [14]. Moreover, in healthy humans, *α*-syn expression was detected in PBMCs, T lymphocytes, B lymphocytes, natural killer cells, and monocytes [15]. It has been also reported that *α*-syn plays an important role in the development and function of T lymphocytes [16].

Sirtuin 3 (SIRT3) is a member of the silent information regulator 2 (Sir2) protein family. It is classified as class I SIRT together with SIRT1 and SIRT2, all of which exhibit a deacetylase activity in the presence of oxidized nicotinamide adenine dinucleotide (NAD^+^) [17]. SIRT3, SIRT4, and SIRT5 are all located in mitochondria, and SIRT3 is considered an enzyme of the mitochondrial matrix [18]. In addition, SIRT3 catalyzes the deacetylation of two enzymes important for antioxidative protection, namely, SOD2 and isocitrate dehydrogenase 2 (IDH2). The activation of SOD2 and IDH2 mediates the antioxidant effect of SIRT3 [19]. The neuroprotective action of SIRT3 was also reported in neurodegenerative disorders such as Alzheimer's disease [20] and recently in an experimental model of PD [21]. SIRT3 expression was also evident in PBMCs [22].

Thus, PBMCs, which can be obtained with minimally invasive procedures, may serve as a model for studying the relationship between neurodegeneration and immunity in patients with neurodegenerative disorders. Moreover, the metabolic pathways of PBMCs occur also in the nervous system (e.g., glutamate pathways). In addition, in pathological (inflammatory/immune) conditions, the PBMCs can penetrate into the central nervous system through the blood-brain barrier (BBB) and cause local effects. In an animal model of experimental autoimmune encephalitis, the migration of PBMCs across the BBB was reported with the involvement of chemokines [23, 24]. The intracellular domain of brain endothelial intercellular adhesion molecule 1 (ICAM-1 or CD54) is required for the transmigration of T lymphocytes through the BBB [25]. Moreover, the penetration of activated monocytes through the BBB is mediated by signal transduction via the tumor necrosis factor (TNF) receptor 1 (TNFR1) [26]. In an animal model of neurodegeneration, PBMC transmigration was also demonstrated [27].

Based on these previous findings, we aimed to study the mitochondrial respiration in intact PBMCs in relation to the expression and activity of SIRT3 in patients with movement disorders.

## 2. Material and Methods

### 2.1. Patients

The study included 57 patients with movement disorders. Idiopathic PD was represented by 38 patients (age 60 ± 9 years), while 19 patients were diagnosed with AP (age 63 ± 10 years), including PSP (*n* = 6), CDB (*n* = 3), MSA (*n* = 9), and DLBD (*n* = 1).

The following data were extracted from the patients' medical history: (1) duration of disease, (2) presence of motor fluctuations and dyskinesias, (3) comorbidities, and (4) treatment. All patients underwent standard neurological examination. Patients with symptoms of any acute disease (e.g., inflammation, infection, and metabolic imbalance) or exacerbation of chronic comorbidities were excluded from the study. In addition, 10 healthy, age-matched volunteers were enrolled in the study as controls. Written informed consent was obtained from all the participants. The study protocol was accepted by the Bioethics Committee of Poznan University of Medical Sciences.

### 2.2. PBMC Isolation and Preparation

PBMCs were isolated from ethylenediaminetetraacetic acid (EDTA) blood via density gradient centrifugation (Histopaque, Sigma-Aldrich). The isolated fractions were supplemented with protease inhibitor cocktails (Sigma-Aldrich; 1 : 200 vol/vol) on ice. The cell number was counted in Bürker's chamber and the volume corresponding to 10^6^ cells was applied for respirometry. Cell viability was tested using the Trypan blue method.

For the SIRT3 analyses, PBMCs were incubated for 30 min in lysis buffer (150 mM NaCl, 50 mM Tris-HCl (pH = 8.0), 5 mM EDTA, and 1% vol/vol Triton X100) with protease inhibitor cocktails (Sigma-Aldrich; 1 : 200 vol/vol) on ice. Subsequently, the lysate was centrifuged for 10 min at 20,000 ×*g*. Protein contents in the PBMC lysates were evaluated using the Lowry method [28].

### 2.3. High-Resolution Respirometry

Mitochondrial respiration was analyzed in intact PBMCs according to the ROUTINE, LEAK, electron transfer system (ETS), and residual oxygen consumption (ROX) protocol [29] using a high-resolution respirometer (Oxygraph-2k; Oroboros Instruments, Innsbruck, Austria). Briefly, a total of 10^6^ PBMCs were added to the respirometer chamber after 10 min of stabilization at 37°C and incubated with continuous stirring at a speed of 750 rpm. The data were subsequently collected with the application of DatLab software 6.1.0.7 (Oroboros Instruments, Innsbruck, Austria).

**Figure 1:**
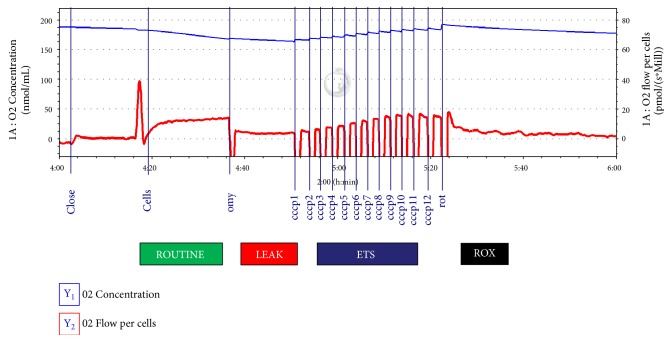
ROUTINE, LEAK, electron transfer system capacity (ETS), and residual oxygen consumption (ROX) respiration in intact peripheral blood mononuclear cells (PBMCs). A record of the protocol during high-resolution respirometry recorded with the use of DatLab software.

ROUTINE respiration in intact PBMCs represents the physiological energy turnover in mitochondria at intracellular nonsaturating adenosine diphosphate (ADP) levels based only on endogenous substrates, that is, without external supply of substrates. The LEAK state is induced by the administration of oligomycin (2 *μ*L, 2 mg/mL concentration), which is an inhibitor of ATP synthase, that is, the phosphorylation system. The LEAK state is a resting, oxygen flux, which compensates proton leak, proton slip, and circulation across the inner mitochondrial membrane. The ETS capacity is the maximum oxygen flux induced by an optimal administration of uncoupler (carbonyl cyanide m-chlorophenyl hydrazone, CCCP, 5 *μ*L steps, 1 mM concentration). The ROX state was subsequently induced by administration of rotenone (10 *μ*L, 2.5 *μ*M concentration), an inhibitor of complex I, thus leading to nicotinamide adenine dinucleotide (NADH) oxidation. The ROX state reflects active oxidative side reactions after ETS inhibition (Figure 1).

### 2.4. SIRT3 Expression and Activity

The activity of SIRT3 was measured by means of a fluorometric method using the SIRT3 Activity Assay Kit (Abcam, Cambridge, UK). Three types of blanks were analyzed: one without patient sample, one without enzyme recombinant, and one without NAD^+^, and the respective volumes were substituted with double-distilled water. The standard curve for SIRT3 expression in PBMCs was fitted using analyses of 0.5, 1.0, 1.5, and 2.0 *μ*g of recombinant SIRT3. Deacetylase activity of SIRT3 was evaluated using kinetic measurements at 2 min intervals using a microtiter plate fluorometer (FLx800, BioTek Instruments, USA) with an excitation set at 340–360 nm and emission at 440–460 nm during 30 min. The rate of reaction was calculated while the reaction velocity remained constant. The expression of SIRT3 was evaluated by means of end-point measurements based on the calibration curve using recombinant SIRT3 (Abcam, Cambridge, UK). The expression of SIRT3 in PBMCs was represented as *μ*g/mg of proteins and deacetylase activity as units (U)/mg of proteins.

### 2.5. Substantia Nigra Echogenicity and Measurement of the Diameter of the 3rd Ventricle

Transcranial ultrasound measurements of the substantia nigra echogenicity and the diameter of the 3rd ventricle were performed using ALOKA Prosound Alpha 7 device according to standard procedures.

### 2.6. Statistics

Statistical analyses were performed using the licensed MedCalc software version 16.8.4-64 bit. First, the distribution of the variables was tested using the D'Agostino-Pearson test. Subsequently, the results with a Gaussian distribution were analyzed using Student's *t*-test for independent variables and expressed as mean ± standard deviation (SD), while those with a non-Gaussian distribution were analyzed with Mann–Whitney *U* test and expressed as median (interquartile range). The significance level (alpha level) was set at or below 0.05.

## 3. Results

The results indicated that ROUTINE respiration in PBMCs was reduced in patients with movement disorders compared with healthy controls (Table 1). Oxygen fluxes in LEAK and ROX states were also lower in patients with movement disorders than in controls (Table 1). No differences in mitochondrial respiration in PBMCs were observed between patients with PD and AP (Table 2).

**Figure 2:**
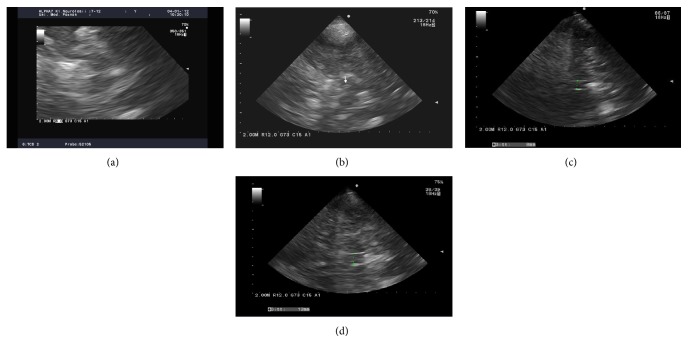
Ultrasound examination of the substantia nigra in a representative control (a) and patient with Parkinson's disease (PD) (b). The arrow in (b) indicates hyperechogenicity. The vertical green dotted line indicates normal 3rd ventricle in the healthy participant (c) and dilated 3rd ventricle in the patient with PD (d).

Among patients with movement disorders, 59% showed substantia nigra hyperechogenicity (Figure 2(b)) with an area of 0.22 ± 0.02 cm^2^ (mean ± SD). The diameter of the 3rd ventricle was 7.2 ± 2.5 mm in patients with movement disorders, while the reference value was 4.0 ± 0.74 mm [30].

ROUTINE respiration was inhibited in patients with PD exhibiting substantia nigra hyperechogenicity (Figure 2) compared with controls (Table 3). The oxygen flux in the LEAK state was lower in patients with PD with substantia nigra hyperechogenicity than in healthy controls (Table 3).

We have not found differences in the expression of SIRT3 protein in PBMCs from patients with movement disorders and controls; however, the deacetylase activity was significantly lower in patients with movement disorders (Table 1). The SIRT3 deacyetylase activity was downregulated in patients with PD compared with controls, while no differences between PD and AP were observed (Table 2). Furthermore, there was no association between SIRT3 expression in PBMCs and the substantia nigra hyperechogenicity (Table 3). Interestingly, the SIRT3 deacetylase activity was inhibited in patients with movement disorders without substantia nigra echogenicity (Table 3).

ROUTINE respiration and ROX correlated negatively with the diameter of the 3rd ventricle measured using ultrasound (*r* = −0.3820, *P* = 0.0373 and *r* = −0.3969, *P* = 0.0330, resp.) in patients with movement disorders. ROX correlated negatively with the age of patients with movement disorders (*r* = −0.2724, *P* = 0.0463).

In patients without hyperechogenicity of the substantia nigra, LEAK respiration correlated negatively (*r* = −0.636, *P* = 0.0353) with SIRT3 expression in PBMCs.

**Figure 3:**
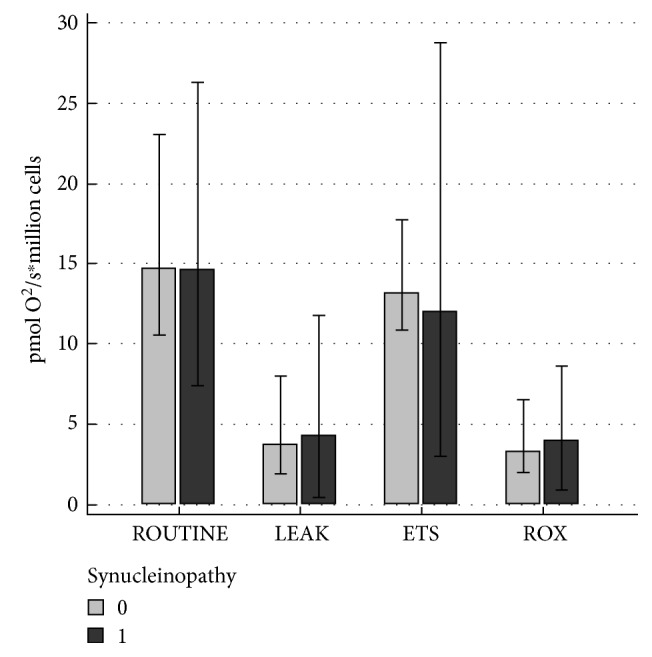
Comparison of the mitochondrial respiration in peripheral blood mononuclear cells (PBMCs) in patients classified to the group of synucleinopathy-related movement disorders (1) and without synucleinopathy (0).

In our group of patients, 48 (84%) had movement disorders, which were classified as synucleinopathies (PD, MSA, and DLBD). There were no differences in the mitochondrial respiration between patients with synucleinopathies and patients without *α*-syn pathology (Figure 3). Moreover, there was no difference in the SIRT3 activity or its expression in patients with movement disorders related to synucleinopathy (66.8; 41.9–111.8 U/*μ*g protein and 35.5; 23.1–58.7 *μ*g/mg protein) compared with patients without synucleinopathy (104.8; 30.9–146.2 U/*μ*g protein; median, interquartile range, *P* = 0.6652 and 36.6; 28.5–57.7 *μ*g/mg protein, median, interquartile range, *P* = 0.7913). In movement disorders without *α*-syn pathology (*n* = 9, PSP and CDB), we have found a positive correlation (*r* = 0.714; *P* = 0.0465) between ROUTINE respiration and SIRT3 expression in PBMCs and positive correlation between ROX and SIRT3 expression in PBMCs (*r* = 0.833; *P* = 0.0102). We have not found correlation between SIRT3 activity or SIRT3 expression and duration of movement disorders. There was also no association between SIRT3 activity or SIRT3 expression in early and late stages of movement disorders.

**Figure 4:**
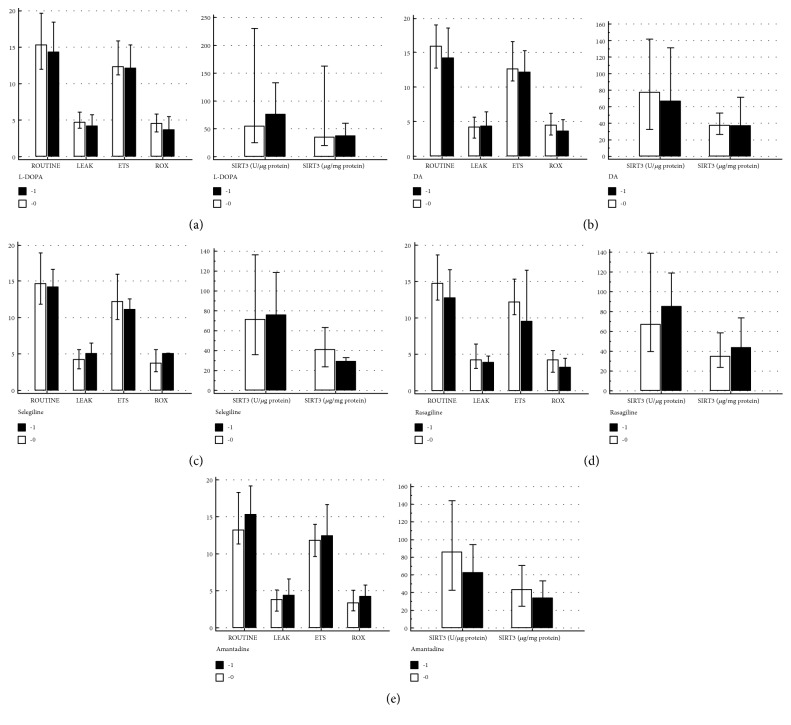
The mitochondrial respiration results and SIRT3 expression/activity in patients with movement disorders (*n* = 57) with respect to treatment (1) and no treatment (0) using (a) levodopa (L-DOPA; A0, *n* = 6; A1, *n* = 51); (b) dopamine (DA) receptor agonists ropinirole and pramipexole (B0, *n* = 20; B1, *n* = 37); (c) selegiline (C0, *n* = 52; C1, *n* = 5); (d) rasagiline (D0, *n* = 47; D1, *n* = 10); and (e) amantadine (E0, *n* = 28; E1, *n* = 29). The results are expressed as median (interquartile range).

No differences in mitochondrial respiration in intact PBMCs were observed between treated (1) and untreated (0) patients with movement disorders, regardless of the treatment, which consisted of levodopa, dopamine receptor agonists (ropinirole, pramipexole), selegiline, rasagiline, or amantadine (Figure 4). Similarly, no differences in SIRT3 expression/activity were noticed between treated and nontreated patients with movement disorders (Figure 4).

## 4. Discussion

The present study indicated that primary neurodegenerative movement disorders, particularly idiopathic PD, are associated with impaired mitochondrial respiration in intact PBMCs and with reduced SIRT3 activity.

The impairment of mitochondrial function, particularly in association with complex I downregulation, has been extensively studied in brains of patients with PD [31, 32]. Moreover, mitochondrial pathology was also suggested in other movement disorders. In patients with PSP, a decreased activity of o-ketoglutarate dehydrogenase in the frontal cortex has been reported, while the activities of complexes I and IV remained unaffected [33]. MSA in animals is induced by chronic administration of 3-nitropropionic acid, which inhibits mitochondrial complex II [34]. In Lewy body disorders, rare variants in the mitochondrial-targeting sequence (MTS) domain of the coiled-coil-helix-coiled-coil-helix domain containing 2 protein encoding genes (*CHCHD2*) have been identified [35].

Mitochondria were also studied in non-neural tissues and cells. Shinde and Pasupathy [36] reported disturbances in mitochondrial respiration in lymphocytes in patients with PD on the basis of the activity measurements of succinate cytochrome c reductase (complexes II and III), rotenone-sensitive NADH cytochrome c reductase (complexes I and III), citrate synthase, succinate dehydrogenase (complex II), and cytochrome c oxidase (complex IV). The downregulation of rotenone-sensitive NADH cytochrome c reductase (complexes I and III) and cytochrome c oxidase (complex IV) was observed in parkinsonian patients and was associated with a decreased ratio of complexes I and III to complexes II and III [36]. Other studies on the mitochondria of lymphocytes revealed an inhibition of complex II [37] and complex IV [38] activities, while studies on isolated mitochondria showed normal activities [39]. In leukocytes isolated from patients with PD, the activities of complexes I and IV were decreased; however, in patients with *PARKIN* gene mutations, the activity of complex IV was unchanged [40].

The studies on the platelets' mitochondria contributed conflicting data. For example, normal mitochondrial function was observed [41–44], along with downregulated activities of complex I [37, 45–49], complex II + III [48], or complex IV [47].

In skin fibroblast cultures from patients with PD, the inhibition of complex V activity was reported, although the levels of oxidized, reduced, and total coenzyme Q10 and activities of Cu/Zn- and Mn-SOD, glutathione peroxidase, and catalase remained unchanged [50].

Our results showing a decreased ROUTINE respiration in PBMCs of patients with movement disorders are in concordance with conclusions of most of the abovementioned studies. However, we have used a high-resolution respirometry instead of the analyses of enzymatic activity evaluations. Analyses in our study were performed in intact PBMCs and in real time. Reduced ROUTINE respiration in intact PBMCs reflects a decreased energy turnover in the mitochondrial and downregulated metabolic state. The decreased LEAK state in intact PBMCs from our patients with movement disorders indicates that the oxygen flux, which compensates the proton leak, proton slip, and cation circulation across the inner mitochondrial membrane, is impaired, particularly in patients with idiopathic PD. The reduced ROX observed in intact PBMCs from patients with movement disorders in our study indicates a rather downregulated nonmitochondrial respiration and, to some extent, a decreased non-ETS respiration. These results may also be the consequence of the depletion of endogenous substrates. All our observations indicated a mitochondrial dysfunction in patients with movement disorders and particularly PD.

ROUTINE and LEAK respirations were also decreased in patients with hyperechogenicity of the substantia nigra, while ROUTINE and ROX correlated negatively with the diameter of the 3rd ventricle measured using ultrasound. Thus, the impairment of mitochondrial respiration is associated with ultrasound measures of neurodegeneration in patients with movement disorders, particularly with PD. Substantia nigra hyperechogenicity was observed in 91% of the patients with PD [51] and in only 9% of patients with AP like MSA and PSP [52]. It is still not clear what might be the cause of the substantia nigra hyperechogenicity in a transcranial ultrasound of the midbrain. The most likely explanation, which was confirmed in both animals and postmortem studies, speculated that this sign is the result of the accumulation of iron within this brain structure [53] and microglial activation [54]. This is consistent with the hypothesis on the role of iron metabolism disturbances in neurodegenerative diseases, including PD [55]. Iron bound to transferrin enters the mitochondria and reaches complex I via the transferrin/transferrin receptor 2 pathway. The accumulation of transferrin in the mitochondria has been reported in a rotenone model of PD [56]. Moreover, the inhibition of the mitochondrial aconitase activity led to iron accumulation in mitochondria and such a process is exacerbated by ROS production [57]. The studies that contributed to the understanding of the association between mitochondrial metabolism, iron accumulation, and substantia nigra hyperechogenicity support our observations of impaired mitochondrial function and ultrasonological measures of neurodegeneration.

SIRT3 activity, but not SIRT3 protein content in PBMCs, was significantly reduced in our cohort of patients with movement disorders and particularly those with PD. In recent years, a number of studies reported a protective role of SIRT3 in neurodegenerative disorders. In a cellular model of PD, SIRT3 was demonstrated to catalyze deacetylation leading to the activation of mitochondrial enzyme citrate synthase [58]. SIRT3 maintains mitochondrial integrity by stimulating a variety of enzymes including SOD2 and glutathione peroxidase, which is involved in ROS scavenging [59]. The expression of SIRT3 together with SIRT5 was stimulated by *α*-syn in PC12 cells, while SIRT1 expression was decreased [60]. Moreover, SIRT3 participates in the antioxidant response in activated microglia [61]. Despite the decreased SIRT3 activity in our patients, we have found a positive correlation between SIRT3 protein expression and mitochondrial respiration in PBMCs. This may indicate that SIRT3 is also involved in the protection of the mitochondrial respiration in patients with movement disorders. From the sirtuin gene family, only SIRT2 was shown as a modulator of proteotoxicity associated with PD [62]. The deacetylase activity of SIRT3 reduces the impact of subcellular stress on mitochondria via the stabilization of ETS and reduction of oxidative stress [63]. Such posttranslational modifications of SIRT3 activity can be a future therapeutic target.

In Chinese patients with PD, polymorphisms in SIRT1 gene, such as g.69644133C>G, g.69644213G>A, and g.69644351G>A, have been identified [64]. However, in the Spanish PD population, no association between *SIRT* gene polymorphisms and PD was found [65]. A single-nucleotide polymorphism in SIRT5prom2 was reported to have an impact on brain aging and risk of neurodegeneration [66].

We found no differences neither in mitochondrial respiration nor in SIRT3 expression or activity in subgroups of patients with movement disorders treated with specific drugs. The main limitation of our present study was its open-label, observational nature. However, particularly noticeable was the absence of differences in the analyzed parameters between levodopa-treated and levodopa-naive patients. The safety of levodopa therapy was discussed for decades, but the drug remains a golden standard in the treatment of idiopathic PD in clinical practice. Nevertheless, levodopa toxicity has been also reported. For example, levodopa injured mitochondria *in vitro* in cell cultures via pro-oxidative processes [67]. Studies in animal models of PD also revealed toxic effects of levodopa [68]. On the other hand, levodopa showed protective [69] and antioxidant effects [70]. Thus, our observation is in concordance with the latter studies.

To conclude, idiopathic PD is associated with impaired mitochondrial respiration in intact PBMCs and reduced SIRT3 activity. The activity of respiratory states in intact PBMCs correlated with the SIRT3 activity and neurodegeneration measures in transcranial ultrasound examination of patients with PD. No differences in mitochondrial respiration in PBMC or SIRT3 activity were observed depending on the treatment.

## Figures and Tables

**Table 1:** Mitochondrial respiration and sirtuin 3 (SIRT3) expression/activity in patients with movement disorders and in controls. Results are presented as mean ± standard deviation (ROUTINE, ROX) and median (interquartile range for LEAK, ETS, and SIRT3). All outcomes of mitochondrial respiration are measured in pmol O2/s^∗^10^6^ cells. SIRT3 protein expression is represented as *μ*g/mg protein. SIRT3 activity is represented as U/*μ*g protein.

	Controls *n* = 10	Patients with movement disorders *n* = 57	*P*
ROUTINE (pmol O_2_/s^∗^10^6^ cells)	19.3 ± 4.4	**15.3 ± 4.5**	**0.0237**
LEAK (pmol O_2_/s^∗^10^6^ cells)	6.02 5.37–6.78	**4.2** **2.98–5.68**	**0.033**
ETS (pmol O_2_/s^∗^10^6^ cells)	13.34 11.22–17.39	12.21 9.84–15.79	0.4168
ROX (pmol O_2_/s^∗^10^6^ cells)	5.7 ± 1.6	**4.2 ± 2.0**	**0.0486**
SIRT3 expression (*μ*g/mg protein)	63.9 20.5–115.4	35.4 23.2–57.9	0.4309
SIRT3 activity (U/*μ*g protein)	181.17 60.9–344.6	**71.40** **38.7–132.9**	**0.0178**

**Table 2:** Mitochondrial respiration and sirtuin 3 (SIRT3) expression/activity in patients with Parkinson's disease (PD), atypical parkinsonism (AP), and controls (C). Results are presented as mean ± standard deviation (ROUTINE and ROX) and median (interquartile range for LEAK, ETS, and SIRT3). All outcomes of mitochondrial respiration are measured in pmol O2/s^∗^10^6^ cells. SIRT3 protein expression is represented as *μ*g/mg protein. SIRT3 activity is represented as U/*μ*g protein.

	Controls (C) *n* = 10	Patients with Parkinson's disease (PD) *n* = 38	Patients with atypical parkinsonism (AP) *n* = 19	P-PD versus C	P-AP versus C	PD versus AP
ROUTINE (pmol O_2_/s^∗^10^6^ cells)	19.3 ± 4.4	**15.2 ± 4.1**	15.7 ± 5.3	**0.0143**	0.644	0.6518
LEAK (pmol O_2_/s^∗^10^6^ cells)	6.02 5.37–6.78	**4.1** **2.97–5.41**	4.24 3.57–6.95	**0.0138**	0.2665	0.3089
ETS (pmol O_2_/s^∗^10^6^ cells)	13.34 11.22–17.39	12.03 9.61–15.45	13.1 10.56–17.23	0.4171	0.5050	0.4828
ROX (pmol O_2_/s^∗^10^6^ cells)	5.7 ± 1.6	**3.75** **2.61–5.52**	3.92 ± 1.98	**0.0430**	0.0754	0.9604
SIRT3 expression (*μ*g/mg protein)	63.9 20.5–115.4	37.1 22.2–59.5	35.4 24.0–57.9	0.4309	0.5952	0.9429
SIRT3 activity (U/*μ*g protein)	181.17 60.9–344.6	**66.8** **43.9–111.8**	77.4 30.9–146.2	**0.0195**	0.0570	0.8312

**Table 3:** Comparison of mitochondrial respiration and sirtuin 3 (SIRT3) expression/activity in a subgroup of patients with movement disorders with substantia nigra hyperechogenicity and in controls. Results are presented as mean ± standard deviation (ROUTINE and ROX) and median (interquartile range for LEAK, ETS, and SIRT3). All outcomes of mitochondrial respiration are measured in pmol O2/s^∗^10^6^ cells. SIRT3 protein expression is represented as *μ*g/mg protein. SIRT3 activity is represented as U/*μ*g protein.

	Controls (C) *n* = 10	Substantia nigra hyperechogenicity present (SNH+) (59%)	Substantia nigra hyperechogenicity absent (SNH-) (41%)	*P* (SNH+) versus C	*P* (SNH−) versus C	*P* (SNH+) versus (SNH−)
ROUTINE (pmol O_2_/s^∗^10^6^ cells)	19.3 ± 4.4	**14.1 ± 3.6**	15.9 ± 4.9	**0.007**	0.1332	0.3768
LEAK (pmol O_2_/s^∗^10^6^ cells)	6.02 5.37–6.78	**3.4** **2.1–5.2**	4.4 3.6–5.5	**0.0203**	0.0822	0.6603
ETS (pmol O_2_/s^∗^10^6^ cells)	13.34 11.22–17.39	12.1 9.9–15.2	12.2 9.3–14.3	0.1947	0.3465	0.9167
ROX (pmol O_2_/s^∗^10^6^ cells)	5.7 ± 1.6	4.1 ± 2.0	4.1 ± 2.5	0.0801	0.1337	0.9484
SIRT3 expression (*μ*g/mg protein)	63.9 20.5–115.4	35.5 24.0–47.8	42.7 19.4–60.1	0.9606	0.6666	0.9606
SIRT3 activity (U/*μ*g protein)	181.17 60.9–344.6	90.1 37.3–141.2	**66.6** **34.7–137.3**	0.6570	**0.0364**	0.6570
